# Progress and outlook of Sn–Pb mixed perovskite solar cells

**DOI:** 10.1186/s40580-023-00371-9

**Published:** 2023-06-16

**Authors:** Hyemin Lee, Seok Beom Kang, Sangwook Lee, Kai Zhu, Dong Hoe Kim

**Affiliations:** 1 Department of Materials Science and Engineering, Korea University, Seoul, 02841 Republic of Korea; 2 School of Materials Science and Engineering, Kyungpook National University, Daegu, 41566 Republic of Korea; 3 Chemistry and Nanoscience Center, National Renewable Energy Laboratory, Golden, CO 80401 USA

**Keywords:** Sn–Pb mixed perovskite, Narrow bandgap, Low bandgap, Mixed tin–lead, Perovskite solar cells, Tandem solar cells

## Abstract

Organic–inorganic hybrid perovskites have revolutionized solar cell research owing to their excellent material properties. Most previous research has been done on Pb-based perovskites. Recently, efforts to discover a Pb-free or Pb-less perovskite material with an ideal bandgap ranging 1.1–1.3 eV have led researchers to investigate Sn–Pb mixed perovskites. Sn–Pb mixed perovskites have a bandgap of ~ 1.25 eV, which is suitable for high-efficiency single-junction and perovskite/perovskite tandem solar cells. Moreover, the Pb content of Sn–Pb mixed perovskites is 50–60% lower than that of Pb-based perovskites, partially mitigating the Pb toxicity issue. However, incorporating Sn^2+^ into the crystal structure also causes various drawbacks, such as inhomogeneous thin film morphologies, easy oxidation of Sn^2+^, and more vulnerable surface properties. Researchers have made substantial progress in addressing these challenges through improvements in compositional design, structural optimization, precursor design, and surface treatments. In this review, we provide a comprehensive overview of the progress in Sn–Pb mixed perovskite solar cells. Furthermore, we analyze the key variables and trends as well as provide an outlook for future directions in the research on Sn–Pb mixed perovskites.

**Graphical Abstract:**

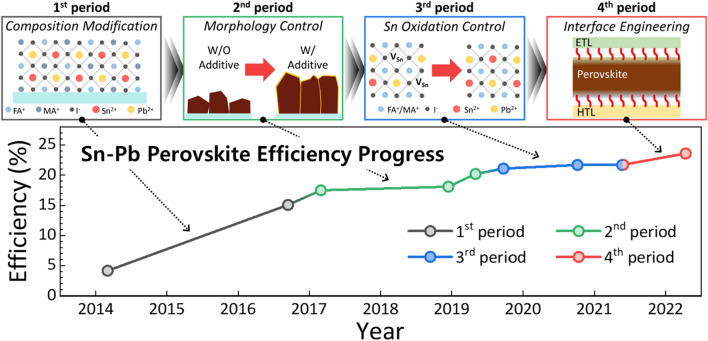

## Introduction

Many researchers have been studying organic and inorganic composite perovskites as next-generation solar cells for more than a decade owing to their excellent material properties [1–10]. Substantial contributions from researchers with various backgrounds have resulted in the rapid development of perovskite solar cells (PSCs), which have demonstrated an increase in the certified efficiency from 14.1% in 2014 to 25.7% recently; notably, such progress is unprecedented in the field of polycrystalline solar cells [4, 5]. Additionally, the advantages associated with the bandgap tuning properties of perovskites have enabled the development of tandem solar cells, including silicon/PSCs [6, 7], CIGS/PSCs [8], and perovskite/PSCs [9]. Silicon/PSCs have recently achieved certified efficiencies of up to 32.5% [10]. Altogether, the development of organic–inorganic hybrid perovskite materials has revolutionized solar cell research.

**Table 1:** Performance and characteristics of low bandgap PSCs based on Sn–Pb mixed perovskites

Year	Period	*V*_OC_ (V)	*J*_SC_ (mA cm^−2^)	FF (%)	PCE (%)	Long-term stability (Measure conditions)	Features	Ref
2014	1st	0.42	20.04	50.00	4.18	–	First NIR PSCs report	[20]
2014	1st	0.73	14.16	64.01	7.37	–	Found that the non-linear bandgap behavior in Sn and Pb mixed perovskites	[21]
2016	1st	0.82	22.44	78	14.35	94% for 30 days (30–40% RH^a^ in N_2_, shelf lifetimes, unencapsulated)	First use of PEDOT:PSS as HTL and p-i-n structure in Sn–Pb mixed low bandgap PSCs	[22]
2016	1st	0.795	26.86	70.6	15.08	–	Development of a new manufacturing method combining FASnI_3_ and MAPbI_3_	[15]
2016	1st	0.69	22.84	65	10.24	–	Using a new fullerene derivative as an electron transport layer	[23]
2016	1st	0.74	26.7	71	14.1	85% for 50 min (50 ± 5% RH ambient air, MPP^b^ tracking, unencapsulated)	Improving the stability and performance of Sn–Pb mixed PSCs by mixing Cs and FA	[24]
2017	1st	0.78	25.69	70	14.01	99% for 1 month (In N_2_, shelf lifetimes, unencapsulated)	Addition of ascorbic acid to suppress oxidation of Sn–Pb mixed perovskite	[25]
2017	2nd	0.857	28.7	71.3	17.5	94% for 33 days (Ambient air, shelf lifetimes, encapsulated)	Modifying the thickness of Sn–Pb mixed perovskite by controlling the precursor concentration	[53]
2017	2nd	0.736	23.5	79	13.7	80% for 7 days (20–25 ℃, 30–50% RH, shelf lifetimes, unencapsulated)	Addition of C_60_ to the precursor to reduce pinholes in the perovskite thin film	[26]
2018	2nd	0.77	26.53	78	15.93	–	Reduce trap state by adding [SnF_2_(DMSO)]_2_ complex instead of SnF_2_	[27]
2018	2nd	0.841	29	74.4	18.1	–	Addition of chloride to increase grain size, crystallinity, and carrier mobility	[28]
2019	2nd	0.842	30.3	79.2	20.2	88% 100 h (MPP tracking, encapsulated)	The defect reduction and carrier lifetime increase through the addition of GASCN	[16]
2019	2nd	0.81	33.14	76	20.4	–	Reducing lattice strain and trap density by Cs ion incorporation	[29]
2019	2nd	0.79	24.95	72	14.03	80% for 30 days (In N_2_, shelf lifetimes, unencapsulated)	Improving crystallinity through recrystallization via MACl post-treatment	[59]
2019	3rd	0.831	31.4	80.34	21.1	–	Suppression of oxidation of Sn^2+^ by addition of Sn metal	[17]
2019	3rd	0.85	30.2	79	20.3	–	Increasing electron diffusion length by adding CdI_2_	[30]
2019	3rd	0.843	30.58	80.34	20.7	95% for 2 months (In N_2_, shelf lifetimes, unencapsulated)	Suppression of oxidation of Sn^2+^ by addition of Sn powder	[31]
2019	3rd	0.72	24.3	82.6	14.4	–	Improving charge extraction through GABr post-treatment	[32]
2020	3rd	1.02	26.61	76	20.63	85% for 1000 h (50–60% RH, shelf lifetimes, unencapsulated)	Reducing defects through the addition of GABr	[33]
2020	3rd	0.85	31.6	80.08	21.7	80% for 30 h (Dry air box, < 20% RH, shelf lifetimes, unencapsulated)	Suppression of oxidation of Sn^2+^ and defect passivation by FSA addition	[18]
2020	3rd	0.78	32.5	71.8	18.2	92% for 120 min (~ 25 ℃, MPP tracking)	Reduction of Sn_y_Pb_(1-y)_I_2_ aggregation by Cs substitution	[34]
2020	3rd	0.81	31.4	75.2	19.1	90% for 1000 h (~ 25 ℃ in N_2_, shelf lifetimes)	Reducing defects and improving crystallinity through the addition of IMBF_4_	[35]
2021	3rd	0.85	27.89	73.13	17.33	87% for 1080 h (In N_2_,shelf lifetimes, unencapsulated)	Mitigating *V*_OC_ loss through the addition of PEAI	[36]
2021	3rd	0.825	30.2	80.1	20.0	80% for 750 h (45 ℃ in N_2_, MPP tracking, encapsulated)	Improving crystallinity and reducing residual stress by adding SnCl_2_·3FACl complex	[37]
2021	4th	0.86	31.86	80	21.74	–	Reduction of surface defects through EDA treatment	[38]
2021	4th	0.834	30.6	79.41	20.27	90% for 350 h (Shelf lifetimes, encapsulated)	Photoelectrical and topological effects of SnF_2_	[39]
2022	4th	0.88	32.77	80	23.3	90% for 1026 h (RT in N_2_, constant 1 sun)	Using 2PACz/MPA bilayer as new hole transport layer	[19]
2022	4th	0.89	32.5	82	23.6	80% for 200 h (In N_2_, MPP tracking, unencapsulated)	Reduction of interfacial defects by adding GlyHCl and surface treatment with EDAI_2_	[40]
2022	4th	0.912	30.73	78.7	22.1	82% for 1830 h (30–35 ℃ in N_2_, MPP tracking, unencapsulated)	Defect passivation and faster charge extraction by adding PEAI and GASCN	[44]

^a ^*RH* relative humidity

^b^*MPP* maximum power point

Although most of the previous PSC research was conducted on Pb-based perovskite materials, multiple research groups have currently been investigating a Pb-free or Pb-less perovskite material with an ideal bandgap range (1.1 to 1.3 eV) for achieving a higher theoretical efficiency as well as potentially reduced toxicity compared to that of Pb-based perovskites [9, 11–13]. Importantly, Sn–Pb mixed perovskite is the only alternative, among various others, that has shown promising performance close to that of Pb perovskite. Sn–Pb perovskites can reach a bandgap of 1.25 eV due to bandgap bowing, a phenomenon in which the bandgap shrinks when Sn and Pb are mixed. This bandgap range is ideal for photovoltaic cells, enabling the development of high-efficiency single-junction devices and perovskite/perovskite tandem solar cells. Additionally, Sn–Pb mixed perovskites have a 50–60% lower Pb content than conventional Pb perovskites, thereby resolving the toxicity issues. However, changes in the electrical and chemical properties caused by the incorporation of Sn^2+^ into the crystal structure of Sn–Pb mixed perovskites have resulted in various undesirable effects [14]. For example, new problems that differ from those of Pb-based perovskites have emerged, such as poor extinction coefficient properties due to less Pb, inhomogeneous thin-film morphologies due to the fast crystallization kinetics of Sn^2+^, high defect concentrations, and poor carrier lifetimes due to the easy oxidation of Sn^2+^ to Sn^4+^. To address these challenges, many researchers have attempted various efforts since they were first reported in 2014, and the results have been remarkable [15–19].

This review focuses on the progress in addressing these challenges in Sn–Pb mixed PSCs compared to the progress in the development of Pb-based PSCs. Specifically, we provide a comprehensive overview of the key research trends and variables, including the composition of Sn–Pb mixed perovskites, device structure, precursor design, and process conditions, through quantitative and qualitative analyses of published reports. Furthermore, we introduce and analyze representative research results that have led to improvements in the performance and stability of solar cells. Finally, we summarize the potential of Sn–Pb mixed perovskites and provide an outlook for future research.

## Trends in Sn–Pb mixed perovskite research

**Fig. 1:**
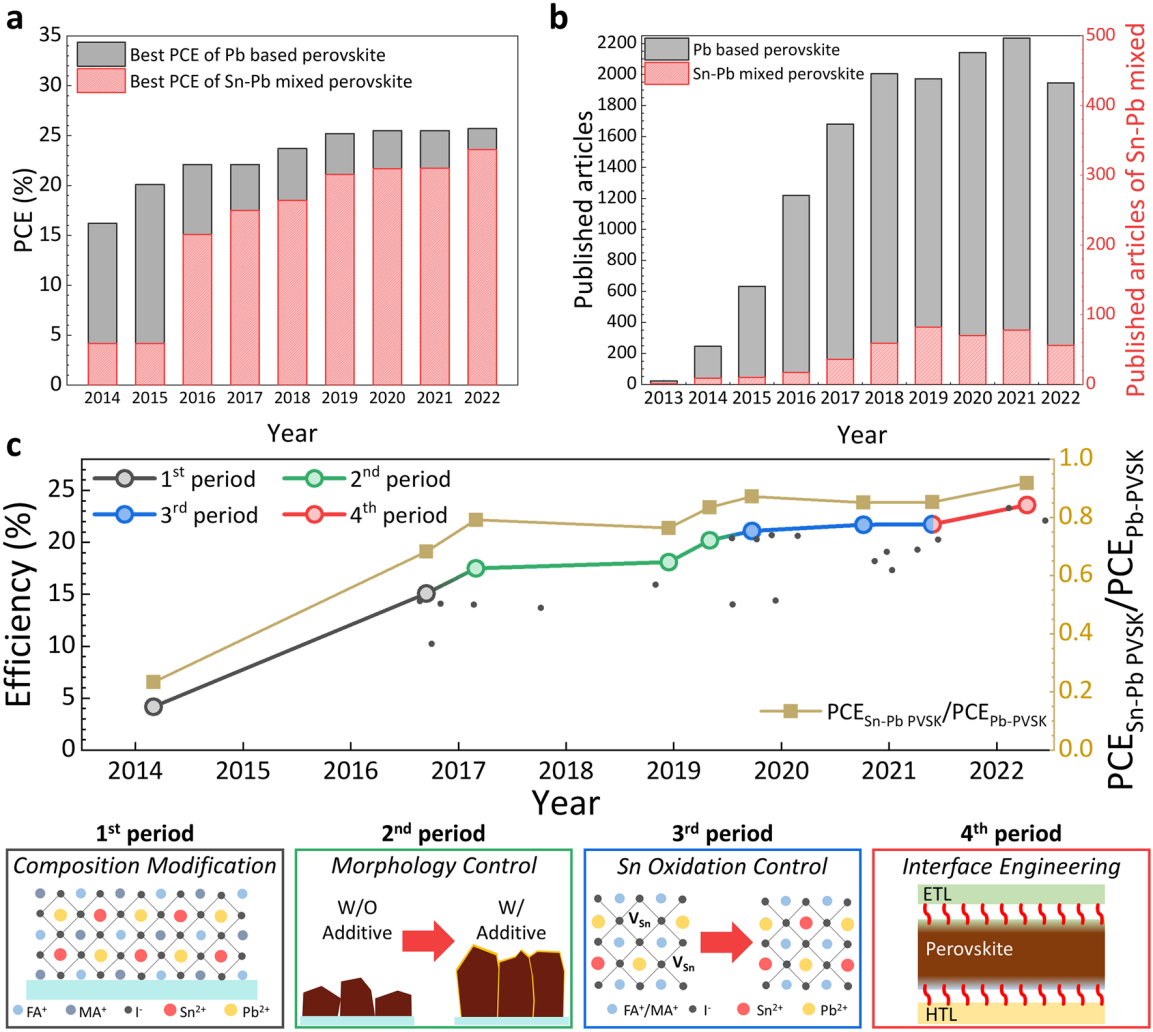
Comparison of efficiency and research progress between Pb-based and Sn–Pb mixed based perovskite solar cells (PSCs).** a** Comparison of best efficiency by year of Pb-based and Sn–Pb mixed PSCs. **b** Number of publications by year of Pb-based and Sn–Pb mixed PSCs. **c** The reported efficiencies (left axis) of Sn–Pb mixed PSCs (dots on a graph). The larger, colorful dots above the line represent the highest efficiencies in each period and are important findings that distinguish research trends. The relative efficiency degree between Pb-based and Sn–Pb mixed PSCs (right axis). The schematic diagrams below show representative variables for each corresponding period of the research trend [15–40]

Figure 1a shows the difference in efficiency between the Sn–Pb mixed PSCs and the Pb-based PSCs. The first reported Sn–Pb mixed PSCs had an efficiency of only approximately 25% of that of Pb-based PSCs [20]. However, continuous improvements since 2019 have increased the efficiency of Sn–Pb mixed PSCs to 80–90% of that of Pb-based PSCs [16]. Most recently, Sn–Pb mixed PSCs have achieved an impressive efficiency of 23.6% [40], which is 90% more than the efficiency of Pb-based PSCs. Figure 1b shows the number of published research articles on Pb-based and Sn–Pb mixed PSCs from 2013 to 2022, reflecting the degree of interest among researchers worldwide. Since 2018, more than 2000 papers on Pb-based PSCs have been published each year, whereas the number of published papers on Sn–Pb mixed PSCs has been much lower, averaging only 70 papers per year. Nevertheless, the number of publications on Sn–Pb mixed PSCs has been increasing steadily. Despite the small number of publications, Sn–Pb mixed PSCs have demonstrated photoconversion efficiencies (PCEs) comparable to those of Pb-based PSCs due to the significant quality improvement of the Sn–Pb mixed perovskite. Notably, the small number of publications on Sn–Pb-based perovskites does not reflect their lack of popularity but rather indicates a barrier for entry into research. Thus, this review aims to discuss the strategies for overcoming the barriers in Sn–Pb perovskite research as well as provide directions for further studies and the development of Sn–Pb perovskite-based devices.

Figure 1c shows the trend in the development of Sn–Pb mixed PSCs over time. The detailed parameters into these efficiency trends ar shown in Table 1. We can classify the study of Sn–Pb mixed PSCs into four periods based on the comparison of their PCEs with those of Pb-based PSCs (less than 70%, 70–85%, 85–90%, and more than 90%). This review discusses the changes in the research trends of the key variables during each period.

The 1st research period of Sn–Pb mixed PSCs can be considered the initial stage, during which the possibility of developing B-site-modified perovskites was mainly explored by mixing various transition metals, such as Ge–Pb [13], Ge–Sn [41], and Sn–Pb [20]. Among the different candidates for incorporation into the B-site of a perovskite structure, Sn–Pb mixed perovskites exhibited the most promising potential as an alternative light-absorbing layer for lowering the bandgap values. Thus, the focus during this research period was to determine the possibility of replacing Pb with Sn at the B site of the perovskite structure. Several groups have reported on Sn–Pb mixed perovskites [15, 42, 43] following their first report by Shuzi Hayase's group, confirming their potential as light absorption materials [20]. However, Sn–Pb mixed PSC still showed a relatively 30% lower efficiency than that of Pb-based PSC (15.08% and 22.1% for Sn–Pb mixed and Pb-based PSCs, respectively), indicating the need for further research to improve the quality of Sn–Pb mixed perovskite materials.

As shown in Fig. 1c, during the 2nd research period, researchers focused on methods for improving the quality, morphology, and thickness of Sn–Pb mixed perovskite polycrystalline thin films. Incorporation of Sn^2+^ ions into the perovskite structure resulted in a lower light absorption coefficient than that of Pb-based perovskites. Additionally, the significant difference in the crystallization kinetics between Pb^2+^ and Sn^2+^ ions caused poor film uniformity and deteriorated the perovskite crystallinity. To address these issues, researchers have designed precursors based on additives such as chlorides [28], GASCN [16], and CdI_2_ [30]. These reported precursor strategies could mitigate most of the aforementioned issues, improving the quality of Sn–Pb perovskite films. However, Sn–Pb mixed perovskite films still exhibited insufficient charge transport characteristics to efficiently extract the photogenerated charge compared with Pb-based perovskites. Many researchers have attributed this limitation to the formation of Sn vacancies due to the insufficient antioxidation of Sn^2+^ ions.

During the 3rd research period, researchers focused more actively on preventing the oxidation of Sn^2+^ ions. The primary methods reported for inhibiting oxidation involved the use of additives that act as antioxidants. Consequently, the Sn–Pb mixed PSC eventually achieved an efficiency of over 21%, reducing the relative efficiency difference with Pb-based PSC to less than 20% (21.7% and 25.5% for Sn–Pb mixed and Pb-based PSCs, respectively). During this period, numerous reports have indicated that Sn–Pb mixed perovskite films exhibited increased carrier diffusion lengths and lifetimes. Recently, the carrier lifetime of Sn–Pb mixed perovskites has increased to more than 9 μs [44].

After the development of various antioxidant additives, the main research trends in Sn–Pb mixed perovskites shifted to surface defect control during the 4th research period [38]. Despite the decrease in Sn vacancies, the increase in efficiency remained relatively stagnant, as discussed in the previous section. Consequently, many scientists achieved performance improvements by improving the interfacial properties between the perovskite layer and charge transport layers. A commonly used strategy is the adoption of long-chain amine-based surface treatments. The modulated surface of the Sn–Pb perovskite exhibited better charge transfer characteristics at the interface, resulting in an efficiency of more than 23% [19].

Sn–Pb mixed perovskite materials have received less interest from researchers than pure Pb-based perovskites, but significant progress has been made through systematic research in a short period. Consequently, they exhibit excellent solar cell performances and present new possibilities for perovskite-based materials other than Pb-based perovskites. In the following sections, we will discuss the unique material properties of Sn–Pb mixed perovskites as well as the corresponding representative studies carried out in each period. We will discuss the outlook for Sn–Pb-based perovskite materials in the end.

## Material properties of Sn–Pb mixed perovskites

### Sn–Pb bandgap bowing phenomena

**Fig. 2:**
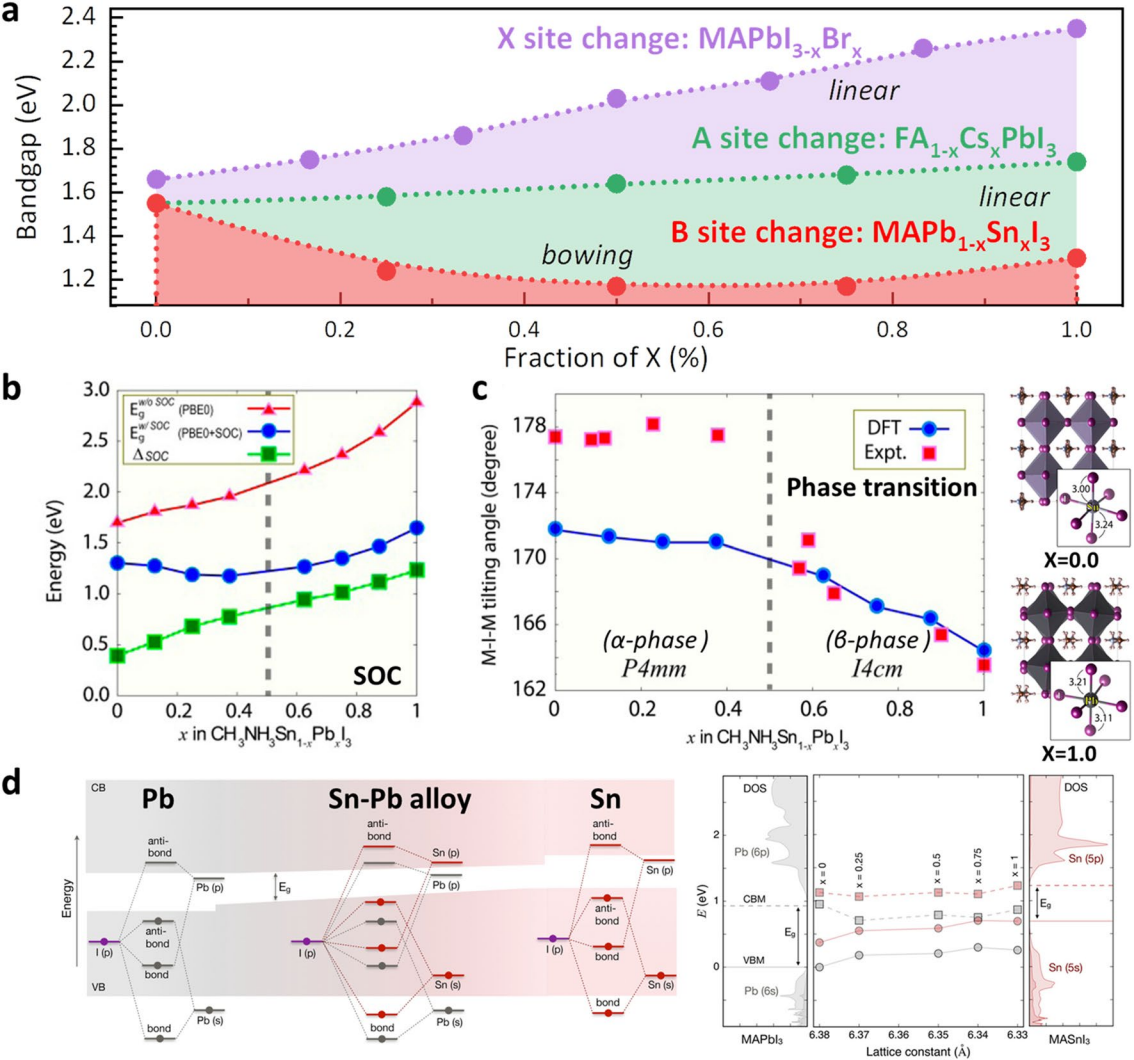
Bandgap structure of Sn–Pb mixed perovskite. **a** The bandgap according to each site change in ABX_3_ perovskite composition [21, 45, 46]. **b** Bandgap trend of MASn_1-x_Pb_x_I_3_ without SOC and with SOC, and the difference in bandgaps without SOC and with SOC,${\Delta }_{\mathrm{SOC}}$. Reproduced with permission from [47], copyright American Chemical Society, 2015. **c** M-I-M tilting angle as a function of x in MASn_1-x_Pb_x_I_3_. Crystal structures of x = 0.0 and 1.0. Reproduced with permission from [47], copyright American Chemical Society, 2015. **d** The bandgap bowing in MA(Pb_1-x_Sn_x_)I_3_ and reduction in the bandgap of alloy compositions by the effect of strain on the band edge energies of pure compositions. Reproduced with permission from [48], copyright American Chemical Society, 2018

Before delving into the detailed research and development stage, it is important to understand the unique characteristics of Sn–Pb mixed perovskites. In general, the compositional changes in semiconductor materials determine the lattice constant according to Vegard's law, which states that the lattice constant has a linear relationship with the bandgap. Figure 2a shows the bandgap change according to the composition variation at each A, B, and X positions in the ABX_3_ perovskite structure. A representative combination of A-sites in the ABX_3_ perovskite structure to form a solid solution is based on formamidinium (FA) and cesium (Cs). For the X site, a halide mixture of iodide, I, and bromide, Br, can be considered a representative example (such as I_(3-x)_Br_x_, where x can vary from 0 to 3). As shown in Fig. 2a, in the case of A-site or X-site solid solution in perovskite, it can be confirmed that the band gap increases linearly as the lattice constant decreases constant according to Vegard’s law [45, 46]. In contrast, when Pb^2+^ and Sn^2+^ ions form a solid solution at the B site in the perovskite structure, bandgap bowing occurs, where the bandgap decreases and then increases according to the ratio of Pb^2+^ and Sn^2+^ ions, which is nonlinear [21]. The origin of bandgap bowing phenomenon has been reported to be divided into two opinions. Prof. Kanatzidis’s group reported that bandgap bowing results from competition between the bandgap decrease due to strong spin–orbit coupling (SOC) and the bandgap increase due to structural distortion [47]. Figure 2b shows the simulated effects of the presence or absence of SOC and ${\Delta }_{\mathrm{SOC}}$ (${E}_{g}^{w/oSOC}-{E}_{g}^{w/SOC}$) on the bandgap change when the B-site Pb composition is increased. When ${\Delta }_{\mathrm{SOC}}$ is applied, the bandgap trend of the MASn_1-x_Pb_x_I_3_ composition changes from a decreasing trend to an increasing trend as the $x$ value increases and shows an inflection point in the $x$ = 0.5 region. Conversely, as shown in Fig. 2c, lattice distortion due to the phase transition, which occurs in the crystal structure according to the increase in the Pb composition, explains the increase in the bandgap. It is well known that the metal-iodide-metal (M-I-M) angle become linear, the greater the overlap of the *p* orbitals, which decreases the bandgap. As the Pb composition increases in Sn-based perovskites, the M-I-M angle loses its linearity and the angle decreases, increasing the bandgap. Figure 2b shows the simulated effect of the presence or absence of SOC, ${\Delta }_{\mathrm{SOC}}$, on the bandgap change when the B-site Pb composition is increased. When ${\Delta }_{\mathrm{SOC}}$ is applied, the bandgap trend of the MASn_1-x_Pb_x_I_3_ composition changes from a decreasing trend to an increasing trend as the $x$ value increases and shows an inflection point at the $x$ = 0.5 region. Conversely, as shown in Fig. 2c, the lattice distortion due to the phase transition, which occurs in the crystal structure with increasing Pb content, explains the increase in the bandgap. In another view, Prof. Stevanovic’s group reported that the nonlinearity of the bandgap, the bandgap bowing phenomenon, is the result of energy mismatch between *s* and *p* orbitals of Pb and Sn which is illustrated in Fig. 2d [48]. To explain in more detail, *s*_Sn_ and *p*_Sn_ orbitals are bound weaker than the corresponding Pb states; therefore, the valence band maximum (VBM) in the alloy is acquired by the interaction between *s*_Sn_ and *p*_I_ orbitals, and the conduction band minimum (CBM) is acquired by the interaction between *p*_Pb_ and *p*_I_ orbitals. Therefore, it was confirmed that the band gap is determined by the CBM derived from MAPbI_3_ and the VBM derived from MASnI_3_. Various mechanisms for bandgap bowing have been suggested, but anyway, it is a unique phenomenon caused by Pb^2+^ and Sn^2+^ ions mixing at the B site of the ABX_3_ perovskite structure.

### Material properties of Sn–Pb mixed perovskites in terms of photovoltaic performance

**Fig. 3:**
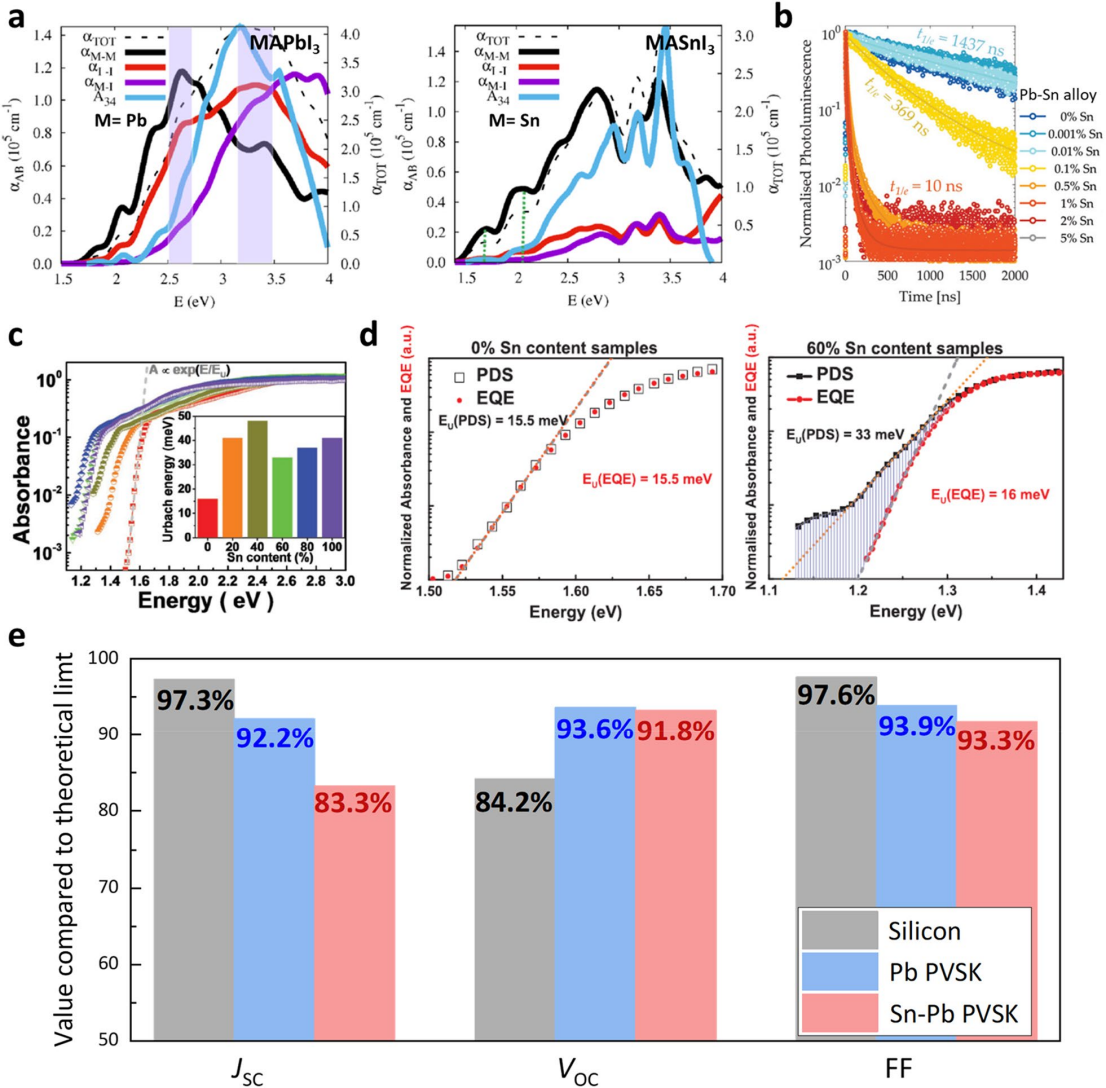
Materials characteristics of Sn–Pb mixed perovskite and their status in terms of photovoltaic properties.** a** Absorption coefficient of MAPbI_3_ and MASnI_3_. Reproduced with permission from [14], copyright American Chemical Society, 2018. **b** PL lifetime for different Sn concentrations in (FA_0.83_Cs_0.17_)(Pb_1-x_Sn_x_)I_3_ materials. Reproduced with permission from [49], copyright Royal Society of Chemistry, 2020. **c** Photothermal deflection spectroscopy (PDS) spectra of MASn_1-x_Pb_x_I_3_ perovskite film with varying Sn concentration. **d** Normalized PDS and EQE spectra of MASn_1-x_Pb_x_I_3_ perovskite. Reproduced with permission from [50], copyright John Wiley and Sons, 2016. **e** Comparison of each parameter of the current highest efficiency silicon, Pb-based and Sn–Pb mixed solar cells as a percentage compared to the theoretical efficiency SQ-limit [10, 40, 51]

The unique properties arising from the mixed Pb^2+^ and Sn^2+^ ions at the B site of the ABX_3_ perovskite structure clearly affect the photovoltaic device properties. Figure 3a clearly shows that the absorption coefficients (${\alpha }_{\mathrm{M}-\mathrm{I}}$ and ${\alpha }_{\mathrm{M}-\mathrm{M}}$) associated with the transition metals in the perovskite decrease when the B site changes from Pb^2+^ to Sn^2+^. This indicates that when Sn^2+^ ions form solid solutions with Pb^2+^ ions, the light absorption properties decrease compared to conventional Pb-based perovskite materials [14]. In addition, as shown in Fig. 3b, even when small amounts of Sn were included in the Pb–Sn alloy, the charge transport properties were drastically reduced [49]. The low light absorption coefficient and inferior charge transport properties significantly affect the actual current density achieved when Sn–Pb mixed perovskites are used as solar cells, resulting in current densities lower than expected from the reduced bandgap effect. Therefore, owing to the nature of the Sn–Pb mixed perovskite, a much thicker and more uniform Sn–Pb mixed perovskite thin film is required to achieve the expected high current density.

Figure 3c shows a graph depicting the calculated Urbach energies of the perovskite materials depending on the Sn content. According to a report by Prof. Sadhanala's group [50], as the Sn content increases, the Urbach energy gradually increases to 48 meV when the Sn content reaches 40%. However, when the Sn content reaches 60%, it decreases to 33 meV before increasing again with increasing Sn content. Figure 3d indicates that, unlike pure Pb-based perovskites, Sn–Pb mixed perovskites exhibit significant differences in photothermal deflection spectroscopy (PDS) technology and photocurrent measurement methods in terms of Urbach energy. This suggests that Sn–Pb mixed perovskites have the potential to have similar Urbach energies as Pb-based perovskites, but they tend to generate many sub-bandgap states owing to the ease of oxidation of Sn^2+^ to Sn^4+^ and other limiting factors. Consequently, many researchers have reported efforts to prevent the oxidation of Sn^2+^ or enhance the quality of thin films in Sn–Pb mixed perovskites to effectively suppress the generation of sub-bandgap states.

Figure 3e shows the achieved percentage against the theoretical Shockley-Queisser limit (SQ limit) of the photovoltaic parameters for the current highest efficiency of Silicon, Pb-based perovskite, and Sn–Pb mixed perovskite photovoltaic devices [10, 40, 51]. The values achieved for each PV parameter allow for the identification of solar cell characteristics based on the properties of the Sn–Pb mixed perovskite materials. Despite the Sn–Pb mixed perovskite having an ideal low band gap, its short-circuit current density (*J*_SC_) value is only approximately 83% of the theoretical *J*_SC_. This is a significant difference compared to the 92% achieved *J*_SC_ for the SQ limit of the Pb PSC. However, it can be confirmed that the reported open-circuit voltage (*V*_OC_) and fill factor (FF) results of Sn–Pb mixed perovskite are comparable to those of the Pb-based perovskite. This is because material properties that are essentially similar to those of Pb perovskite have been well revealed in the photovoltaic properties because of the excellent results of many researchers, such as minimizing the sub-bandgap states. The following section covers the development of Sn–Pb mixed perovskite materials and the improvement of solar cell efficiency based on the results of major studies previously classified by period.

## Trends in the development of Sn–Pb mixed PSCs

### Period I: exploring the possibility of developing Sn–Pb mixed perovskites

**Fig. 4:**
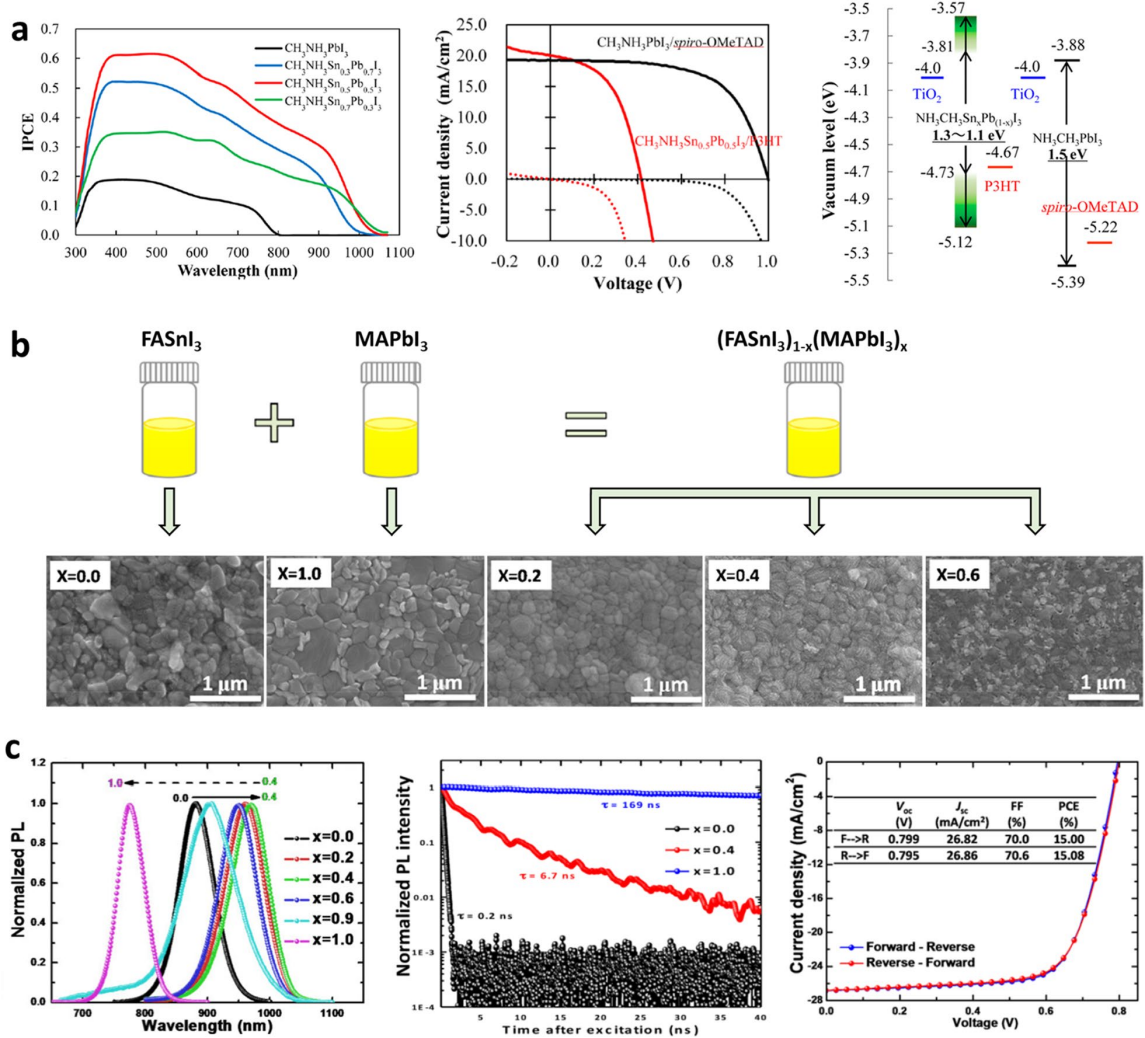
Compositional and crystal structural improvement of Sn–Pb mixed perovskite for solar cells.** a** IPCE curves for MASn_x_Pb_(1-x)_I_3_ PSCs and optimized photovoltaic performance for MASn_0.5_Pb_0.5_I_3_ and MAPbI_3_ PSCs. Energy diagram of MASn_x_Pb_(1-x)_I_3_ perovskite. Reproduced with permission from [20], copyright American Chemical Society, 2014. **b** Schematic view of formation perovskite precursor and SEM images of (FASnI_3_)_1-x_(MAPbI_3_)_x_ perovskite films. **c** Normalized PL spectra of (FASnI_3_)_1-x_(MAPbI_3_)_x_ perovskites, TRPL of (FASnI_3_)_1-x_(MAPbI_3_)_x_ perovskites at x = 0.0, 0.4, 1.0 and *J-V* curves at x = 0.4. Reproduced with permission from [15], copyright American Chemical Society, 2016

In the early stages of research on Sn–Pb mixed perovskites, it was realized that this material had the potential to absorb light up to the near-infrared (NIR) region of 1000 nm or more [52]. As these materials were novel in the PSC field at the time, the primary focus of the research was to develop a suitable perovskite composition and device structure for solar cell applications. The first solar cells utilizing Sn–Pb mixed perovskites were reported by Prof. Hayase’s group [20]. Figure 4a shows the characteristics of solar cells fabricated by replacing Pb^2+^ ions in the B site with Sn^2+^ ions at different ratios based on MAPbI_3_ perovskite. The solar cells were fabricated the conventional n–i–p structure, which is commonly used in Pb-based PSCs. The device comprised a compact TiO_2_ layer/mesoporous TiO_2_ layer-based electron transport layer (ETL), an Sn–Pb mixed perovskite layer as the absorption layer, and a P3HT hole transport layer (HTL). Photovoltaic characteristics appeared when the Sn^2+^ content was below 70%, and the edge of the absorption wavelength in the external quantum efficiency (EQE) widened to 1060 nm. When the Sn^2+^ content was 50%, the edge of the absorption wavelength was maintained at approximately 1050 nm and the highest EQE values were observed. Notably, HTLs play a crucial role in the operation of Sn–Pb mixed PSCs. Specifically, the efficiency of the Sn_0.5_Pb_0.5_-based perovskite was only 0.0016% when spiro-MeOTAD, which is a widely used HTL material, was used as the HTL. However, when the HTL is changed to P3HT, the efficiency increased to 2.37%. This underscores the importance of an appropriate HTL design for efficient hole extraction, as the shallower VBM of Sn^2+^ necessitates a different approach. After optimizing each layer, Hayase’s group reported that MAPb_0.5_Sn_0.5_I_3_-based PSCs exhibited a* V*_OC_ of 0.42 V, FF of 50%, *J*_SC_ of 20.04 mA cm^−2^, and PCE of 4.18%.

Prof. Jen's group was the first to use poly(3,4-ethylenedioxythiophene):poly(4-styrenesulfonate) (PEDOT:PSS) with proper energy alignment with the VBM of a Sn–Pb mixed perovskite, overcoming potential barriers at the interface between the Sn–Pb mixed perovskite and HTL. Finally, they designed an inverted device structure with an ITO/PEDOT:PSS/MAPb_0.75_Sn_0.25_I_3_/PC_61_BM/Bis-C_60_/Ag composition and achieved an efficiency of 14.35% [22]. The structure presented in this work has been applied as a basic framework for Sn–Pb mixed PSC structures to date. Prof. Yan’s group utilized a new precursor that combines FASnI_3_ with the MAPbI_3_ precursor instead of relying solely on the MA-based Sn–Pb mixed perovskite [21, 23], which was mainly used at that time [15]. Scanning electron microscopy (SEM) images of the Sn–Pb mixed perovskite films produced by combining FASnI_3_ precursor and MAPbI_3_ precursor at different ratios are shown in Fig. 4b. The (FASnI_3_)_0.6_(MAPbI_3_)_0.4_ perovskite film exhibited a relatively small grain size; however, the grains formed more uniformly without pinholes.

Figure 4c illustrates the properties of the Sn–Pb mixed perovskite materials, including bandgap bowing and device characteristics utilizing the Sn–Pb mixed perovskite materials. The photoluminescence spectra showed that the luminescence wavelength increased as the Sn content increased, reaching a maximum value at Sn 60%, and then decreasing. The carrier lifetime was measured using time-resolved photoluminescence (TRPL). (FASnI_3_)_0.6_(MAPbI_3_)_0.4_ revealed a carrier lifetime of 6.7 ns. Although this result was significantly shorter than the 169 ns of the carrier lifetime of MAPbI_3_, the lifetime was much improved compared to of 0.2 ns carrier lifetime of FASnI_3_. The researchers then demonstrated the fabrication of PSCs based on (FASnI_3_)_0.6_(MAPbI_3_)_0.4_ perovskite using a p–i–n inverted planar structure with a PEDOT:PSS HTL, which yielded an excellent *J*_SC_ of over 25 mA cm^−2^ and an efficiency of 15.08% [16, 27, 28, 31, 53].

### Period II: improving the quality of Sn–Pb mixed perovskite films

**Fig. 5:**
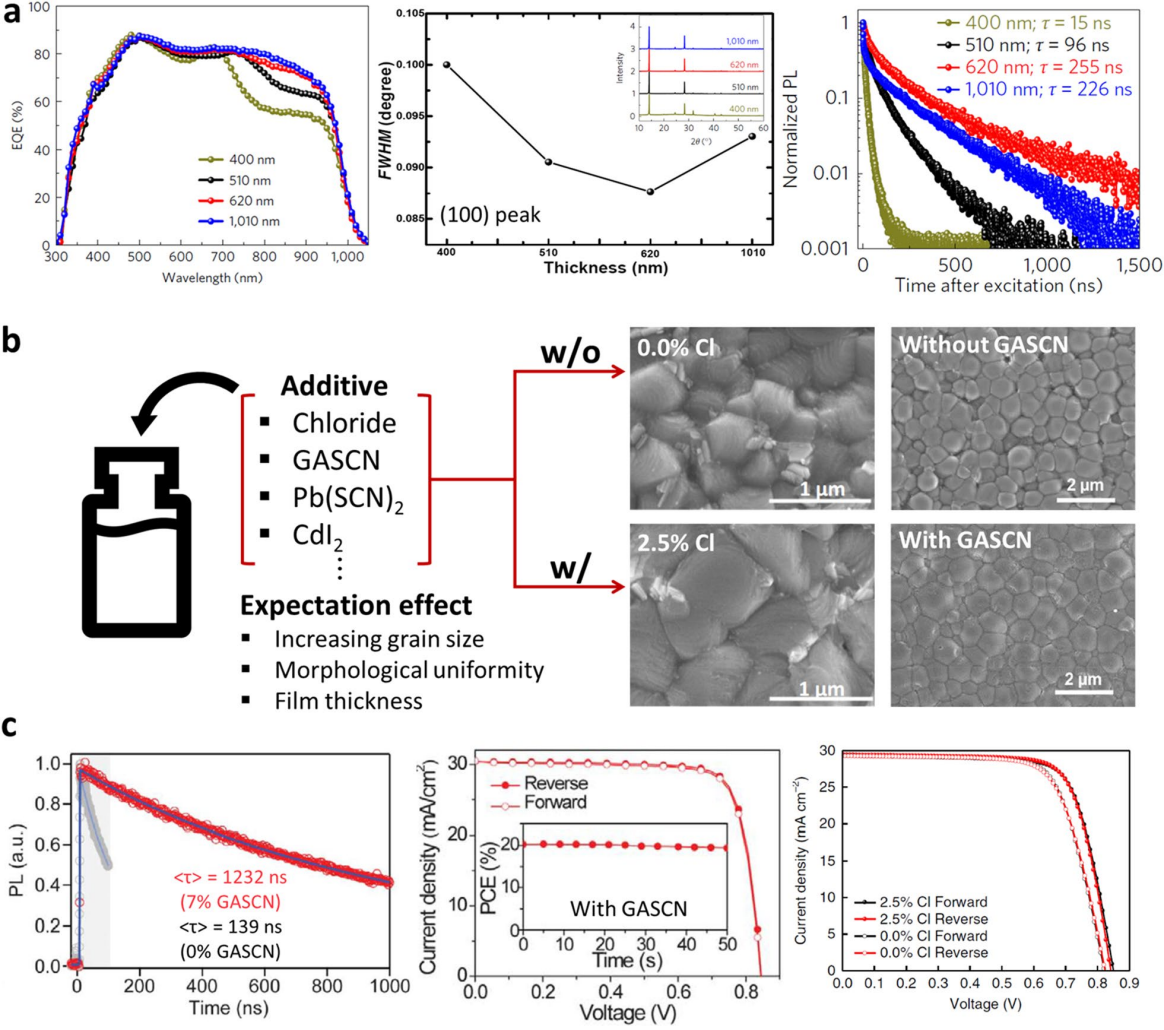
Precursor design via concentration and additives for improving perovskite film quality. **a** EQE curves, XRD FWHM values of (100) peak and carrier lifetime via TRPL of (FASnI_3_)_1-x_(MAPbI_3_)_x_ perovskite with different thickness. Reproduced with permission from [53], copyright Springer Nature, 2017. **b** Various additives effects on the morphological evolution of Sn–Pb perovskite confirmed by SEM. **c** Carrier lifetime via TRPL and *J-V* curves of perovskite solar cells and with and without additives. Reproduced with permission from [28], copyright The American Association for the Advancement of Science, 2019. Reproduced with permission from [16], copyright Springer Nature, 2018

Despite the potential of Sn–Pb mixed perovskite for solar cells with a proper composition and device structure, its efficiency was stagnant at approximately 15%, which lagged behind that of Pb-based PSCs. In particular, the current density, which was considered a strong advantage of the narrow bandgap of the Sn–Pb mixed perovskite, did not meet the expectations. To address this issue, many researchers have explored ways to thicken Sn–Pb mixed perovskite films and improve their morphological uniformity and quality through the development of precise coating conditions or precursor designs using various additives. As shown in Fig. 5a, Prof. Yan's group investigated increasing the spectral response by adjusting the thickness of a Sn–Pb mixed perovskite film [53]. Modulating the concentration of the Sn–Pb mixed perovskite precursor improves the thickness of the perovskite film and increases the EQE in the NIR region by up to 75%. This result demonstrates the potential for high current density of Sn–Pb mixed perovskites and provides a clear direction for future research. However, when the thickness exceeded 620 nm, the crystallinity measured through X-ray diffraction (XRD) and the electron lifetime measured through TRPL tended to deteriorate. As a result, even with a thicker film of 1 μm, there was little improvement in EQE at NIR region. Thus, it is important to not only increase the thickness but also improve the charge-transport properties.

Figure 5b shows the effects of various additives on the film uniformity and crystallinity of the Sn–Pb mixed perovskite and the charge transport properties. Prof. Yan's group reported the effects of chloride additives on Sn–Pb mixed perovskites in terms of their crystal and morphological properties, enabling the formation of a uniform light-absorbing layer over 800 nm thick [28]. Similarly, Dr. Zhu's group improved the morphology of the film surface and passivation of grain boundaries by adding guanidinium thiocyanate (GASCN) and advanced the electrical properties of the film [16]. Figure 5c shows the enhanced device performance when the chloride or GASCN additives were used. The precursor design through additives not only improved the morphological characteristics but also maintained the improved charge transport characteristics, as shown in the TRPL analysis in Fig. 5c. The improved morphological and electrical properties owing to the additives were linked to high photoelectric conversion efficiencies of up to 20%. This made it possible to efficiently utilize the electron–hole pairs photogenerated in the uniformly formed thick absorbing layer.

### Period III: reducing agent of Sn^2+^ ion

**Fig. 6:**
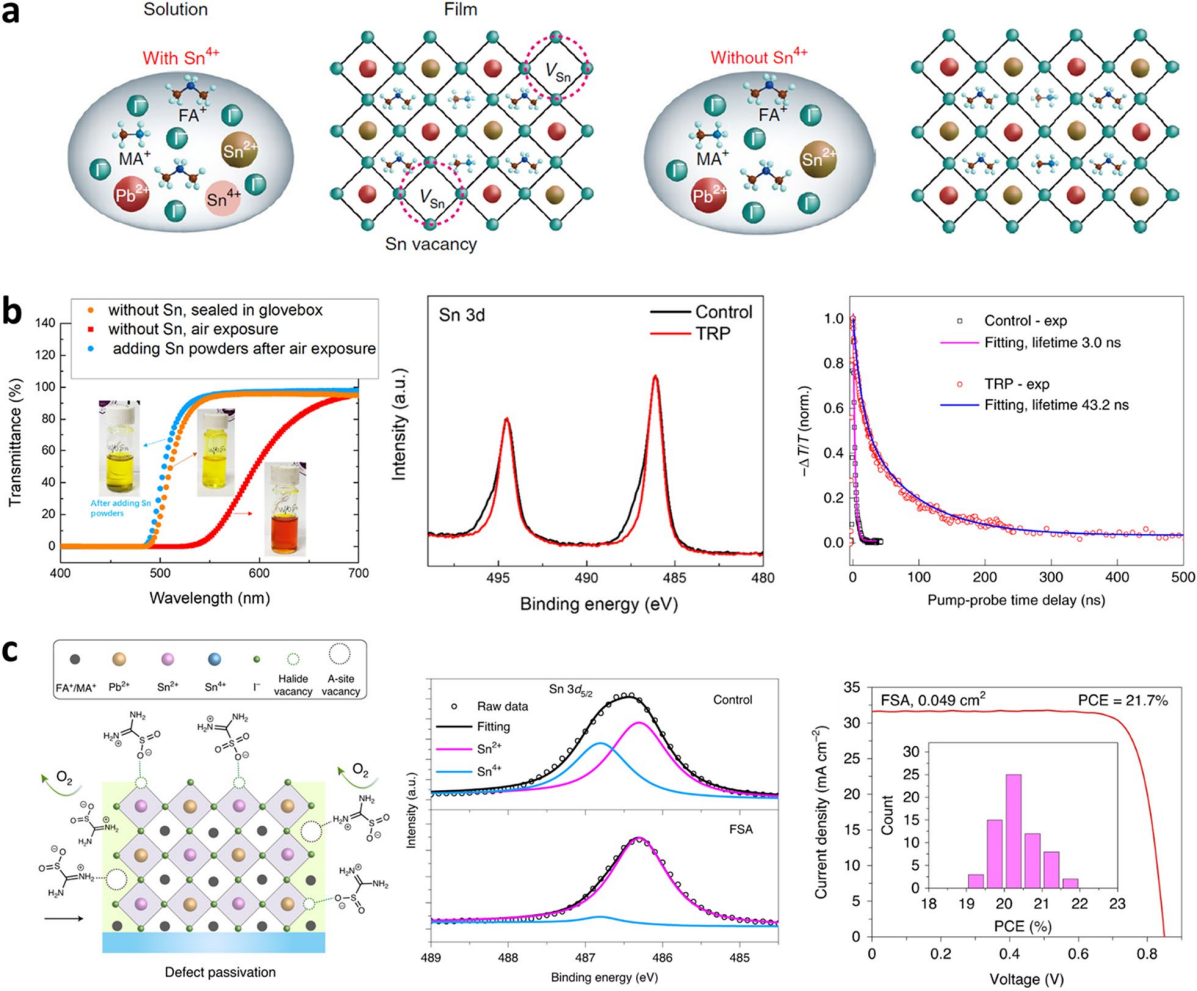
Effect of reducing agents in precursor on Sn–Pb mixed perovskites.** a** Illustration of solution and crystal structure of Sn–Pb-based perovskite with and without reductant. **b** Transmittance, XPS, and TRPL decay without and with Sn metallic powder. Reproduced with permission from [17], copyright Springer Nature, 2019. **c** Schematic diagram of antioxidation and defect passivation with zwitterion FSA. XPS without and with FSA and J-V curve of Sn–Pb mixed PSC with FSA. Reproduced with permission from [18], copyright Springer Nature, 2020

Another inherent problem with Sn–Pb mixed perovskites is the oxidation of Sn^2+^ to Sn^4+^. As illustrated in Fig. 6a, films made from precursors containing Sn^4+^ spontaneously form Sn vacancies in the crystal structure, whereas films made from precursors without Sn^4+^ do not contain Sn vacancies [17]. Let us briefly change the subject for a moment to pure Sn-based perovskites, which were much more vulnerable to Sn^2+^ oxidation, to discuss about early efforts to prevent this. It faces a severe problem of low current density due to the formation of Sn vacancies by oxidation from Sn^2+^ to Sn^4+^. This issue was resolved by Prof. Matthews' group, who conducted a study on reducing Sn^2+^ oxidation by adding SnF_2_, a reductant, to Sn-based perovskite precursor [54]. Let's return to the original topic, Sn–Pb mixed perovskites, SnF_2_ was first applied to Sn–Pb mixed perovskite by Prof. Yan’s group in 2016, and since then, SnF_2_ has been widely used as a basic reductant in research on Sn–Pb mixed PSCs [15–18, 27–36, 38, 39, 44, 53, 55]. As the study on Sn–Pb mixed PSCs progresses, a limitation of the antioxidant effect of the SnF_2_ reductant has been observed, and further research on reductants is required.

As shown in Fig. 6b, Prof. Tan’s group confirmed the limited antioxidant capability of SnF_2_ for Sn^2+^ by changing the color of the solution from yellow to red when a solution containing only SnF_2_ was exposed to air [17]. In contrast, when Sn metallic powder was added to the precursor along with SnF_2_, the color remained yellow. Considering the Frost diagrams for Sn in different oxidation states, the introduction of metallic Sn into a Sn-based perovskite precursor spontaneously results in the reduction of Sn^4+^ to Sn^2+^ via the most favorable reaction Sn + Sn^4+^$\to$ 2Sn^2+ ^ having the negative Gibbs energy. X-ray photoelectron spectroscopy (XPS) analysis of Sn–Pb mixed perovskite shows the Sn 3d_3/2_ (~ 494 eV) and Sn 3d_5/2_ (~ 486 eV) peaks with (TRP, tin-reduced precursor) and without (control) Sn metal powder. The two Sn 3d peaks are a combination of the Sn^2+^ peak with lower binding energy and the Sn^4+^ peak with higher binding energy. In control, the shoulders at the higher binding energy detected in both Sn 3d peaks are caused by Sn^4+^ resulting from the oxidation of Sn ions. However, in TRP, the shoulder was not detected due to the reduction of Sn^4+^, and the Sn^2+^ peak was prominent. Additionally, the TRPL analysis indicated an increase in the carrier lifetime after the addition of metallic Sn powder. Consequently, a Sn–Pb mixed PSC with a PCE of 21.1% was reported.

In 2020, the same group demonstrated another approach to suppress Sn^2+^ oxidation by adding a zwitterionic antioxidant [18]. Zwitterionic additives are known to passivate defects in perovskite films, including both electron-donating defects such as FA/MA vacancies and electron-accepting defects such as halide vacancies and under-coordinated Pb^2+^/Sn^2+^, leading to improved crystal and electronic properties. Formamidine sulfinic acid (FSA) was chosen as the antioxidant zwitterionic additive in this study because the zwitterionic form among the three different tautomers is the most stable in any state of FSA [56]. In addition, FSA in the precursor provided lower volatility than DMSO, which led to the retardation of perovskite crystallization and film formation, promoting crystal homogenization. As shown in Fig. 6c, FSA acts as a strong reductant that returns Sn^4+^ to Sn^2+^ and binds to the FA/MA and halide vacancies. In XPS analysis, the antioxidant effect of FSA was confirmed by the Sn 3d_5/2_ peak. TRPL analysis also confirmed that the carrier lifetime was improved by the addition of FSA. As a result, the *J*_SC_ of the Sn–Pb-based PSCs could reach 31.6 mA cm^−2^. These efforts addressed the existing lack of antioxidant capability, allowing researchers to reliably achieve *J*_SC_ above 30 mA cm^−2^.

### Period IV: interface engineering of Sn–Pb mixed perovskite

Since the formation of Sn–Pb mixed perovskites, many efforts have been made to enhance the quality of perovskite materials, including composition optimization, defect prevention, and oxidation protection. In recent years, research has focused on device-oriented approaches, with attention directed towards improving the interfacial properties between the perovskite and carrier transport layers. The improved interfacial properties can solve the poor charge-transport properties of Sn–Pb mixed perovskites, resulting in significant efficiency improvements. The PCE of the Sn–Pb mixed perovskite reached more than 90% of that of the Pb-based perovskite.

**Fig. 7:**
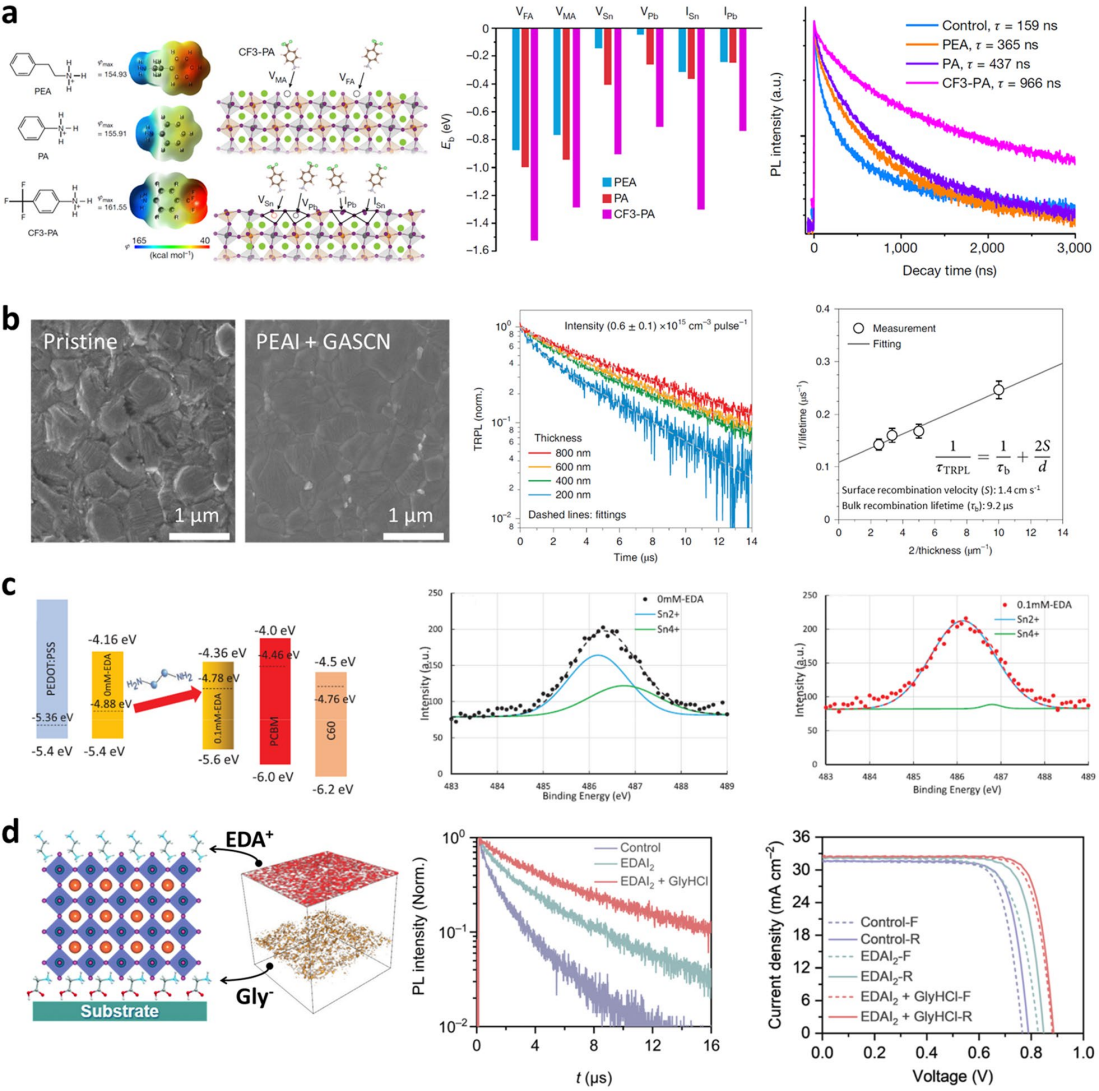
Interface engineering of Sn–Pb mixed perovskites. **a** Schematic diagram of the interaction between ammonium cations and defects. The calculated binding energies of different passivators with various defects. TRPL decays of perovskite with different passivators. Reproduced with permission from [57], copyright Springer Nature, 2022. **b** SEM images of perovskite films without and with mixed PEAI and GASCN additives. Thickness dependence of the TRPL measurements with mixed additives. Surface recombination velocity and bulk recombination lifetime plotted through carrier lifetime with various thickness. Reproduced with permission from [44], copyright Springer Nature, 2022. **c** XPS analysis of perovskite film with and without EDA treatment and energy diagram of PSC with and without EDA treatment. Reproduced with permission from [38], copyright John Wiley and Sons, 2021. **d** Schematic diagram of EDA^2+^ and GlyH^+^ cations forming dipoles at the perovskite interfaces. TRPL decay and *J-V* curves without and with surface treatments. Reproduced with permission from [40], copyright Royal Society of Chemistry, 2022

To improve the interfacial properties, Prof. Tan's group selected aromatic ammonium cations (phenethylammonium (PEA), phenylammonium (PA), and 4-trifluoromethyl-phenylammonium (CF3-PA)) and studied their effects of the molecular electrostatic properties on Sn–Pb perovskite surface defects. The researchers hypothesized that different electrostatic potentials $({\mathbf{\varphi }}_{\mathbf{m}\mathbf{a}\mathbf{x},\mathbf{P}\mathbf{E}\mathbf{A}}<{\mathbf{\varphi }}_{\mathbf{m}\mathbf{a}\mathbf{x},\mathbf{P}\mathbf{A}}<{\mathbf{\varphi }}_{\mathbf{m}\mathbf{a}\mathbf{x},\mathbf{C}\mathbf{F}3-\mathbf{P}\mathbf{A}})$ would give different types of bonding to surface defects. The theoretical results confirmed this hypothesis, showing that CF3-PA has excellent anchoring properties to surface defects compared to the other candidate materials. As shown in Fig. 7a, CF3-PA, which has a high electronegativity and strong electron-withdrawing properties, was applied to the PSC. Through surface defect passivation, the carrier lifetime of the material increased more than 6 times compared to the control (control:159 ns, CF3-PA:966 ns), leading to a *J*_SC_ of 32.9 mA cm^−2^ and an efficiency of 21.9%.

Dr. Zhu’s group improved the surface quality of Sn–Pb mixed perovskites by adding phenethylammonium iodide (PEAI) and GASCN together [44]. The reaction between the two additives formed a quasi-2D structure of PEA_2_GAPb_2_I_7_. The surface morphology was noticeably enhanced owing to the quasi-2D structure of the 3D perovskite, and the under-coordinated B-site defects (Sn^2+^ and Pb^2+^) were passivated. In Fig. 7b, the surface recombination velocity and bulk recombination lifetime were calculated through TRPL measurements by thickness, based on Eq. (1) [58]:

1 $$\frac{1}{{\tau }_{\mathrm{TRPL}}}=\frac{1}{{\tau }_{\mathrm{b}}}+\frac{2S}{d}$$

where τ_TRPL_ is the measured TRPL lifetime, τ_b_ is the bulk recombination lifetime, *S* is the surface recombination velocity, and *d* is the film thickness. The *S* calculated by the equation was very low at 1.4 cm s^−1^, and the bulk recombination lifetime was 9.2 μs. A low value of *S* corresponds to a lifetime of 30 μs for surface recombination velocity, meaning that very minimal recombination occurs on the surface.

To passivate the undercoordinated B-site defects and control the energy level on the surface of the Sn–Pb mixed perovskite, a novel surface treatment was developed. Prof. Hayase’s group demonstrated that treating the top surface of perovskite with ethylenediamine (EDA) can suppress under-coordinated Sn defects and adjust the Fermi energy [38]. As shown in Fig. 7c, Kelvin probe microscopy (KPM) analysis revealed that the Fermi energy of the EDA treated perovskite film ranged from −4.88 eV to −4.78 eV, creating an n-type surface. The changes on the surface are attributed to the increased electron concentration resulting from the electron-donating characteristics of EDA's amine groups. In terms of passivation, the amine groups of EDA were found to attach to the under-coordinated Sn atoms, and XPS analysis confirmed that after EDA treatment, the oxidation of under-coordinated Sn atoms was reduced.

Most recently, efficiencies of 23.6% have been achieved by controlling the interface defects using both additive and surface treatment methods. Prof. Wakamiya's group reported that crystallinity was improved by adding glycine hydrochloride (GyHCl) to the precursor [40]. As shown in Fig. 7d, GlyH^+^ ions aggregated at the interface between the HTL and perovskite layer owing to downward during perovskite film formation, replacing the A-site cations of perovskite. After the formation of the perovskite film, the top surface traps located at the interface between the perovskite and ETL could be reduced through surface polishing and passivation effects through EDAI_2_ surface treatment. Through these two approaches, carrier lifetime increased from 2.8 μs to 4.9 μs which led to a *J*_SC_ of 32.5 mA cm^−2^ and an efficiency of 23.6%.

## Summary and outlook

This article discusses the progress in the development of Sn–Pb mixed PSCs and how they have advanced in comparison to Pb-based PSCs. Researchers have divided the research on Sn–Pb mixed PSCs into four periods based on a comparison of the PCE of Pb-based PSCs and changes in key variable trends over time. Over these four periods, various efforts have been made to address issues related to the incorporation of Sn^2+^ into the crystal structure of Sn–Pb mixed perovskites, including composition, structure, precursor design, and surface treatment. In the first period, researchers focused on confirming the possibility of replacing Pb with Sn at the B site of the perovskite structure. In the second period, efforts were made to improve the quality, morphology, and thickness of Sn–Pb mixed perovskite polycrystalline thin films to overcome their inferior light absorption coefficient. In the third period, researchers focused on preventing the oxidation of Sn^2+^ ions, which led to significant improvements in efficiency. In the fourth period, the focus shifted to surface defect control, resulting in further improvements in efficiency and stability. Consequently, the development of high-quality synthesis processes and surface defect control methods has led to improvements in efficiency and carrier lifetime, and Sn–Pb mixed perovskites exhibited unique opportunities for solar cell applications compared to Pb-based perovskite materials.

However, the *J*_SC_ values of Sn–Pb mixed PSCs are still as low as 83.3% of the theoretical limit of their ideal bandgap. The best reported *J*_SC_ is 32.5 mA cm^−2^, whereas the theoretical *J*_SC_ can reach up to 39.0 mA cm^−2^. To address this problem, various studies have been conducted on Sn–Pb mixed perovskite materials, ranging from conventional precursor designs to surface treatments. However, there have been no studies on optimizing the electron and hole transport layers for the energy-level change that occurs when Sn is incorporated into the perovskite crystal structure. Therefore, it is necessary to explore ETL and HTL optimization for the bandgap structure of Sn–Pb mixed perovskites to take the next step in improving their performance.

Additionally, the low stability of Sn compared to that of Pb is an urgent problem that needs to be solved. Regarding ETL and HTL in terms of stability, PEDOT:PSS, which is currently the most widely used, is considered to have ideal band alignment; however, its strong acidity is known to reduce the stability of Sn–Pb mixed perovskites over time. Therefore, the development of ETLs, HTLs, or interfaces of perovskites that do not exhibit this chemical influence needs to be studied more systematically in terms of stability.

Finally, the development of large-scale deposition and modularization processes is crucial. Unlike Pb perovskite, Sn–Pb perovskite cannot be grafted onto conventional solar cells such as Si and CIGS; therefore, it must be developed as a single-junction device or perovskite/perovskite tandem solar cell. From this perspective, there has been no research on appropriate large-area coating methods and modularization processes.

Although there are still many aspects of Sn–Pb mixed perovskites that need to be studied, the efficiency growth shown in a relatively short period of time with relatively little research attention is quite remarkable. Therefore, if research on Sn–Pb mixed perovskites is systematically conducted in the direction of existing research and this review, it may open up new possibilities for perovskite-based materials beyond Pb-based perovskites in the future.

## Data Availability

Not applicable.

## Abbreviations

PSCs: Perovskite solar cells
PCEs: Photoconversion efficiencies
ETL: Electron transport layer
FA: Formamidinium
Cs: Cesium
SOC: Spin–orbit coupling
M-I-M: Metal-iodide-metal
VBM: Valence band maximum
CBM: Conduction band minimum
PDS: Photothermal deflection spectroscopy
SQ limit: Shockley-Queisser limit
*J*_SC_: Short-circuit current density
*V*_OC_: Open-circuit voltage
FF: Fill factor
NIR: Near-infrared
HTL: Hole transport layer
EQE: External quantum efficiency
PEDOT:PSS: Poly(3,4-ethylenedioxythiophene):poly(4-styrenesulfonate)
SEM: Scanning electron microscopy
TRPL: Time-resolved photoluminescence
XRD: X-ray diffraction
GASCN: Guanidinium thiocyanate
XPS: X-ray photoelectron spectroscopy
TRP: Tin-reduced precursor
FSA: Formamidine sulfinic acid
PEA: Phenethylammonium
PA: Phenylammonium
CF3-PA: 4-Trifluoromethyl-phenylammonium
PEAI: Phenethylammonium iodide
EDA: Ethylenediamine
KPM: Kelvin probe microscopy
GyHCl: Glycine hydrochloride
